# Genome-Wide Analysis of the *12-Oxo-Phytodienoic Acid Reductase* Gene Family in Peanut and Functional Characterization of *AhOPR6* in Salt Stress

**DOI:** 10.3390/plants14101408

**Published:** 2025-05-08

**Authors:** Yifei Mou, Quanxi Sun, Haocui Miao, Juan Wang, Qi Wang, Qianqian Wang, Caixia Yan, Cuiling Yuan, Xiaobo Zhao, Chunjuan Li, Shihua Shan

**Affiliations:** 1 Shandong Peanut Research Institute, Qingdao 266100, China; yifeimou1123@163.com (Y.M.); squanxi@163.com (Q.S.); wangjuan_1984@163.com (J.W.); wqi0309@126.com (Q.W.); wangqianqian@saas.ac.cn (Q.W.); cxyan335@sina.com (C.Y.); yuancl1982@163.com (C.Y.); zhaoxiaoboqd@126.com (X.Z.); peanutlab@163.com (C.L.); 2 Institute of Crop Germplasm Resource, Xinjiang Academy of Agricultural Sciences, Urumqi 830000, China; mc09876@163.com

**Keywords:** peanut, 12-oxo-phytodienoic acid reductase, salt stress

## Abstract

12-oxo-phytodienoic acid reductases (*OPR*s) have been substantiated as pivotal in plant growth and response to biotic and abiotic stresses. However, the functional characterization of *OPR* genes in the peanut genome remains limited. In this study, we identified a total of 20 *OPR* genes in a tetraploid cultivar and two diploid peanut species, categorizing them into two subfamilies, OPRI and OPRII. The gene structure and conserved protein motifs within each subfamily were elucidated. Additionally, our findings indicate an uneven chromosomal distribution of peanut *OPR* genes. Gene duplication events were identified as pivotal in the expansion of the *OPR* gene family. An analysis of cis-acting elements within *OPR* gene promoters revealed the presence of numerous phytohormone- and stress-related cis-elements. Furthermore, peanut *OPR* genes exhibited tissue-specific and stress-inducible expression patterns, underscoring their crucial role in peanut growth and stress response. Additionally, plants overexpressing *AhOPR6* exhibited significantly enhanced resistance to salt stress, and the *AhOPR6*-OE lines demonstrated a higher ability to scavenge reactive oxygen species (ROS). Collectively, these findings offer deeper insights into the roles of peanut *OPR* genes in stress responses, suggesting that *AhOPR6* could serve as a potential candidate gene for improving peanut salt tolerance through genetic transformation.

## 1. Introduction

The cultivated peanut, one of the most important economic oil crops worldwide, provides healthy oil and high-quality protein to the human diet, and it is used as animal feed [1]. Approximately 53% of the world’s peanut production is used as high-quality edible oil, 32% is used for snack peanuts and candies, and about 15% is used as animal feed and in seed production [2]. Peanut plantation is widely carried out all over the world, from semi-arid tropical to subtropical regions [2,3]. The annual worldwide losses in peanut production as a result of various kinds of biotic and abiotic stresses equal approximately USD 3.2 billion [2]. Peanut is a moderately salt-tolerant crop [4]; planting peanut in saline–alkali areas could solve the urgent problem of the need to improve the utilization efficiency of saline–alkali land, which accounts for about 76% of the world’s cultivated land area [5,6].

JA and related derivatives, namely cis-(+)-12-oxo-phytohormone jasmonic acid (cis-OPDA), methyl jasmonate (MeJA), and jasmonoyl-L-isolucine (JA-lle), participate in signal pathways in response to biotic and abiotic stresses, such as low temperature, hot stress, salinity tolerance, wounding, Hessian flies, aphids, and pathogens [7,8]. Besides contributing to the defense response, JA also acts in many plant growth and development processes, including tendril coiling, fruit ripening, pollen maturation, root growth, and seed germination [9,10]. JA biosynthesis mainly occurs in two organelles, chloroplasts and peroxisomes. JA biosynthesis starts from α-linolenic acid (LA) in chloroplast, which is then mediated by lipoxygenase (*LOX*), allene oxide synthase (*AOS*), and allene oxide cyclase (*AOC*) genes to generate 12-oxophytodienoic acid (OPDA) [11]. Afterward, the OPDA is transported into the peroxisome, where it is catalyzed by OPDA reductase (*OPR*) and reduced to 12-oxophytoenoic acid (OPC-8:0). Finally, the OPC-8:0 is converted to JA through three cycles of β-oxidation reaction [7].

12-oxo-phytodienoic acid reductases (*OPR*s) are the key precursors in the biosynthesis of jasmonic acid (JA), and they belong to the old yellow enzyme family (OYE), which contains flavin mononucleotide (FMN) as a non-covalently bound cofactor [12]. This family has several subfamilies in plants [13]. On the basis of the phylogenetic analysis of six green plant groups (green algae, mosses, lycophytes, gymnosperms, monocots, and dicots), *OPR*s can be divided into seven subfamilies. The *OPR*s in monocots are classified into five subfamilies (subs. I-V), while those in dicots are classified into two subfamilies (subs. I-II) [14,15]. In *Arabidopsis thaliana*, which belongs to the dicots, *OPR* can be divided into two groups (OPRI and OPRII) based on substrate specificity [16]. *AtOPR1* and *AtOPR2* (OPRI) enzymes preferentially catalyze (9R,13R)-12-oxophytodienoic acid (9R,13R-OPDA), and *AtOPR3* (OPRII) can convert 9S,13S-OPDA to oxo-2(2′(Z)-pentenyl)-cyclopentane-1-octanoic acid (OPC-8:0) [14,17]. The classification of subfamilies III, IV, and V in rice (a monocot) was thoroughly explained by Li et al. (2011) [14]. The variable regions MVR i and MVR ii affect the three-dimensional (3D) structure of rice *OPR* proteins, which in turn influences the substrate specificity and catalytic activity of different subfamily proteins [14]. In rice, sub. I, sub. II, and sub. V have similar 3D structures, except in the MVR ii region. Sub. I and sub. II exhibit strong or moderate catalytic activity with substrates, while sub. V show weak catalytic activity. The distinction between sub. III and sub. IV, which have similar 3D structures, could be seen in MVR i [14].

To date, the identification of *OPR* genes has been performed in some dicots and monocots, such as *Arabidopsis* [17,18], rice [14], maize [19], tomato [20,21], and wheat [15]. The function of *OPR*s, which belong to subfamilies I and II in monocots (like rice, maize, and wheat) and dicots (*Arabidopsis*, tomato, and cotton), has been well documented; the *OPR*s participate in biotic and abiotic stress in diverse pathways [20,22,23,24,25]. In *Arabidopsis*, *AtOPR1* and *AtOPR2* were highly expressed in the roots and leaves [18], while *AtOPR3* was detected in various tissues, especially in the flowers and anthers [26,27]. It has been reported that OPRI genes participate in some stress responses, such as stress caused by salinity, wounding, low temperatures, and pathogens. In wheat, overexpressing *TaOPR1* (OPRI) could enhance salinity tolerance, and its heterologous expression in *Arabidopsis* promoted ABA synthesis and the ABA-dependent stress-responsive pathway [22]. *TaOPR2* (OPRII), which is involved in the biosynthesis of JA in wheat, and the expression of *TaOPR2* could be induced by wounding, drought, and methyl jasmonate (MeJA) [28]. Additionally, the expression of *TaOPR2* increases after infection with *Puccinia striiformis* f. sp. *tritici* and *Puccinia recondite* f. sp. *Tritici* [28]. In cotton, the transcripts of *AOS*, *AOC*, and *OPR*, along with the increased accumulation of α-linoleic acid, contribute to alleviating salt stress [29]. In rice, the *OsOPR7* gene is crucial for JA synthesis, and *osOPR7* mutants generated through CRISPR/Cas9 show male sterility with lower JA levels, which can be recovered by exogenous MeJA [30]. In maize, the double mutant *OPR7OPR8* exhibited strong developmental defects, delayed leaf senescence, and increased susceptibility to insects and pathogens [23]. Significantly, in plants, only OPR II genes are related to the biosynthesis of JA. Due to the delayed anther filaments and inviable pollen grains, *OPR3* (OPR II subfamily) mutant plants are male sterile in *Arabidopsis*, and this phenotype can be restored by exogenous JA [26]. Silenced *OPR3* in tomato is more susceptible to the necrotrophic pathogen *Botrytis cinerea* and produces fewer seeds than the wild type (WT) [20]. Nevertheless, existing studies focus on the OPRI and OPRII subfamilies primarily in monocot plants and few dicots, and the potential functions of *OPR* genes in peanut remain unclear. Whether they possess a similar pathway in stress response still requires further validation in peanut.

In this study, all of the *OPR* genes in peanut (tetraploid cultivar peanut *Arachis hypogaea* “Tifrunner”, diploid *A. duranensis*, and *A. ipaensis*) were identified using the genome-wide analysis method. Then, the putative 20 *OPR* genes of peanut, 3 genes of *Arabidopsis*, and 10 genes of rice were used to construct a phylogenetic tree. The gene structure, protein conserved motifs, chromosomal locations, cis-acting elements in the promoter, gene duplication, gene ontology, and interactive networks were systematically investigated. Furthermore, the subcellular location of two *OPR* genes, *AhOPR3* and *AhOPR6*, was confirmed. One *OPR* gene (subfamily I) was cloned from the peanut cultivar Huayu71 and named *AhOPR6*. The overexpression of *AhOPR6* in *Arabidopsis* could enhance salt tolerance. The relative expression levels of twelve genes which related to JA and ABA signaling pathways were detected using qRT-PCR to understand the *OPR*s’ potential functions in the ABA- or JA-dependent salt-responsive pathway. The findings of this study highlight the roles of peanut *OPR* genes in peanut growth and development. More importantly, we provide enough evidence that *AhOPR6* enhances salt stress tolerance and present a hypothetical model of *AhOPR6*’s action in improving salt tolerance.

## 2. Results

### 2.1. Identification and Analysis of Peanut OPR Genes

Based on the genomic databases of cultivated and wild-type peanut, 23 *OPR* genes were identified by the HMM profile of *OPR* (accession: PF00724). After confirming the *OPR* domain, it was found that 20 *OPR* genes have the whole domain, and they were used for the following investigation. The coding sequence length of peanut *OPR*s ranged from 339 (*AhOPR5*) to 573 (*AiOPR2*) amino acids. The molecular weights (MWs) of the *OPR* proteins ranged from 37.6 (*AhOPR5*) to 64.4 (*AiOPR2*) kDa, and the theoretical isoelectric points (pI) ranged from 5.52 (*AhOPR5*) to 7.15 (*AhOPR1*). The subcellular localization of most *OPR* proteins (15 out of 20) was predicted to be localized in chloroplast; *AdOPR4* and *AhOPR9* were localized in mitochondria. In addition, two *OPR*s (*AhOPR3* and *AiOPR3*) were predicated to be localized in plasma membrane. The predicted subcellular localizations of *OPR*s need further experimental validation. Detailed information of all of the above *OPR* genes, including the gene ID, protein length, molecular weight, theoretical *pI*, genomic location, and prediction of protein subcellular localization, is shown in Table S1.

### 2.2. Phylogenetic Analysis and Classification of OPR Gene Family

**Figure 1:**
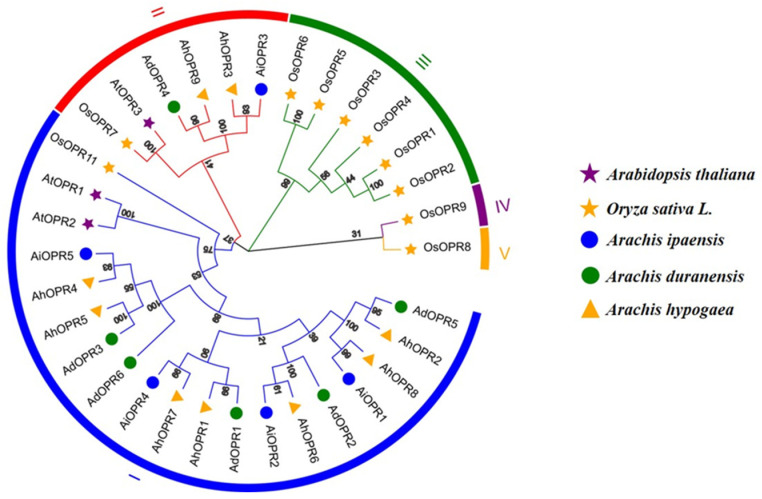
Phylogenetic tree of peanut *OPR* gene family members. Tree was constructed using neighbor-joining (NJ) method with peanut, rice, and Arabidopsis OPR proteins and bootstrap analysis with 1000 replicates.

To investigate the evolutionary relationships of *OPR*s, a maximum likelihood phylogenetic tree (with 1000 bootstraps) was constructed using *Arabidopsis thaliana*, *Oryza sativa* L., and peanut (*Arachis ipaensis*, *Arachis duranensis*, and *Arachis hypogaea*) species. All *OPR*s could be classified into five subfamilies: OPR I, II, III, IV, and V (Figure 1). Twenty *OPR*s from three different peanut species could be divided into two subfamilies, OPR I and OPR II (Figure 1). The classification of peanut *OPR*s is similar to that of dicot plant *Arabidopsis*. Among them, the OPR I subfamily contained the highest number of *OPR* proteins (16 out of 20; 80%), while OPR II contained four *OPR*s, including *AiOPR3*, *AdOPR4*, *AhOPR3*, and *AhOPR9*.

### 2.3. Conserved Motifs and Gene Structure Analysis of Peanut OPR Genes

**Figure 2:**
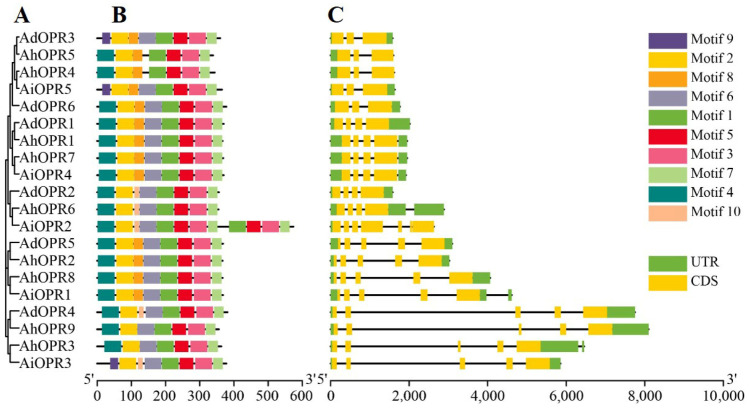
The phylogenetic relationship, gene structure, and protein conserved motif analysis of *OPR* genes in peanut. (**A**) The NJ phylogenetic tree with 1,000 replicates; (**B**) the conserved motifs of OPR proteins; (**C**) the gene structures (exon-intron) of *OPR* genes.

A maximum-likelihood phylogenetic tree (with 1000 bootstraps) was constructed using the multiple sequence alignment (ClustalW) of 20 conserved OPR proteins from peanut (Figure 2A). The MEME tool was used to identify the conserved motifs in peanut *OPR*s. At most, ten conserved motifs, with lengths between 6 and 50 amino acids, were found, showing diversity in each peanut *OPR* (Figure 2B, Table S2). In the OPR I and OPR II subfamilies, motifs 1–3, motif 5, and motif 7 were present in all *OPR*s, and they were the most conserved domains (Figure 2B). In *AiOPR3*, *AiOPR5*, and *AdOPR3*, the position of motif 9 was replaced by motif 4. Four *OPR*s in OPR II lacked motif 8, which was totally associated with the β6 barrel of the protein secondary structures and further affected the 3D structure of *AhOPR3* and *AhOPR9* (Figure S1, Table S2). In the OPR I subfamily, *AhOPR4* and *AhOPR5* lacked motif 6, which reveals that they may have different functions (Figure 2B). Similar motifs in the same subfamily may have similar functions.

To further analyze the gene structure of peanut *OPR* genes, the gene structure features were exhibited by TBtoolsV1.098 (Figure 2C). As shown in Figure 2C, most *OPR*s of peanut contain 3–5 exons. Ai*OPR*2 contained the highest number of exon 7. In addition, we found that the intron numbers and lengths were diverse in different subfamilies (Figure 2C). All the genes in OPR II contained 4 introns, and the second intron was significantly longer than others compared with genes in the OPR I subfamily (Figure 2C). In OPR I, most *OPR*s (approximately 70%) had 2–3 introns, and the others contained more than 4 introns (Figure 2C).

### 2.4. Chromosomal Locations and Synteny Analysis of Peanut OPRs

**Figure 3:**
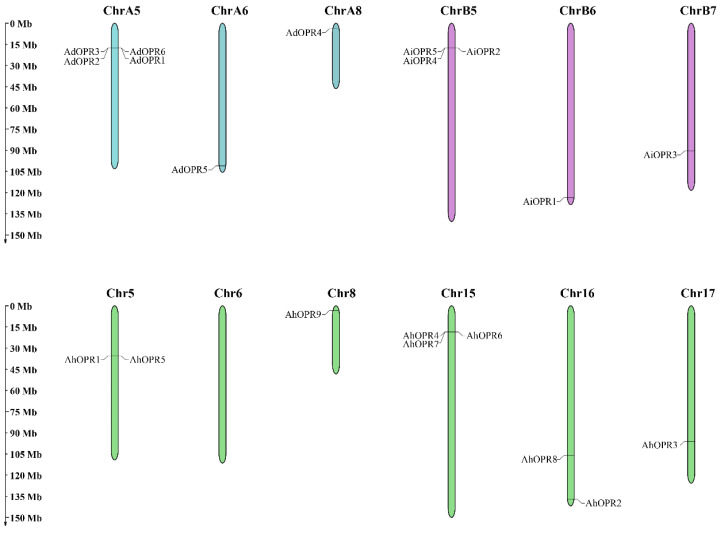
Chromosomal locations of *OPR* genes. Genes from different peanut species are shown in different colors (*Arachis duranensis*: blue, *Arachis ipaensis*: purple, and *Arachis hypogaea*: green).

**Figure 4:**
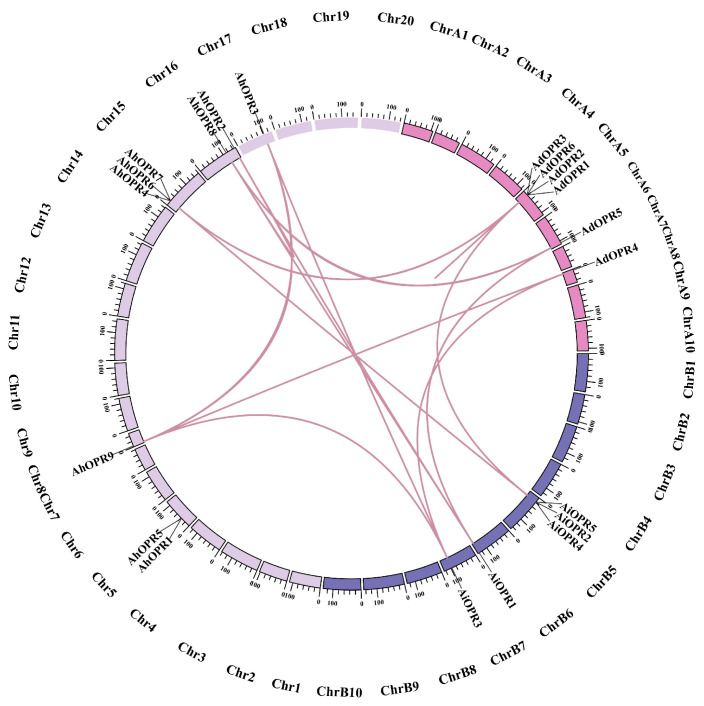
Synteny analysis of *OPR* genes in peanut. Lines highlighted in red indicate duplicated gene pairs of peanut *OPR* genes.

A total of 20 peanut *OPR* genes were unevenly distributed on 40 chromosomes (Figure 3). No genes were found on 29 chromosomes, including chromosomes A1–4, A7, B1–4, B8, 1–4, 7, 9–14, and 18. The maximum number of *OPR* genes appeared in chromosome A5 (4, 20%), and the second largest number of *OPR* genes were found on chromosome B5 (3, 15%) and 15 (3, 15%). There was one gene on chromosomes A6, A8, B6, B7, 8, and 17 (Figure 3). Two *OPR* genes were mapped on chromosomes 5 and 16, respectively (Figure 3). To further study the gene duplication events of peanut *OPR* genes, tandem and segmental duplicated gene pairs were analyzed (Figure 4 and Table S3). There were two groups of tandem duplicated genes, including *AdOPR1* and *AdOPR*2 and *AdOPR*2 and *AdOPR*6, which were linked with red lines. In addition, 14 segmental duplication gene pairs are visualized in Figure 4. The number of segmental duplicated gene pairs was dramatically higher than the tandem duplicated gene pairs; this might be the main reason for the expansion of the *OPR* gene family.

### 2.5. Prediction and Analysis of Cis-Acting Elements in Promoter Sequences of OPR Genes

**Figure 5:**
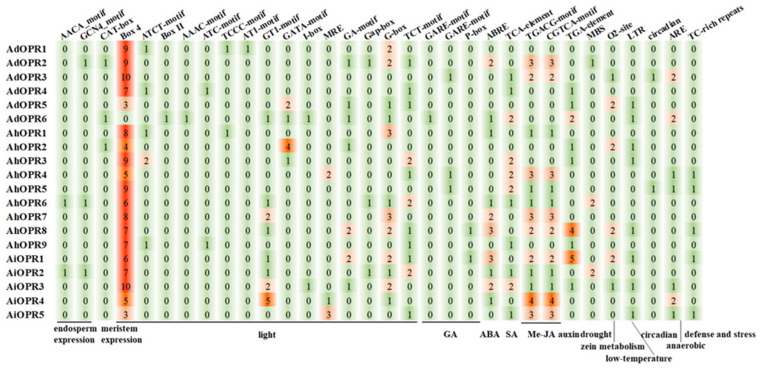
Cis-acting element analysis in promoter regions of peanut *OPR* genes.

To further explore the evolution and functional characterization of peanut *OPR* genes, the 1.5 kb areas upstream from the translational start site of each gene were analyzed. Various cis-acting elements were found in *OPR* promoters (Table S4). As shown in Figure 5, peanut *OPR* promoters contained elements associated with endosperm development, meristem development, light, GA, ABA, SA, MeJA, auxin, drought, zein metabolism, low-temperature, circadian, anaerobic, and defense stresses. The cis-acting elements (Box 4, ATCT-motif, Box II, AAAC-motif, ATC-motif, GT1-motif, GATA-motif, I-box, GA-motif, G-Box, and TCT-motif) related to light were all found in the *OPR* gene promoter. The majority of the *OPR*s included elements responsive to ABA (ABRE), SA (TCA-element), and MeJA (TGACG-motif and CGTCA-motif) (Figure 5). A small number of gene promoters were identified to be involved in responses to drought (MBS), GA (GARE-motif and P-box), auxin (TGA-element), low-temperature (LTR), anaerobic (ARE), and defense stresses (TC-rich repeats). These findings indicated that peanut *OPR* genes might participate in plant growth and development and the response to hormones and diverse stresses.

### 2.6. Expression Patterns of OPR Genes in Different Tissues and in Response to Various Stresses

**Figure 6:**
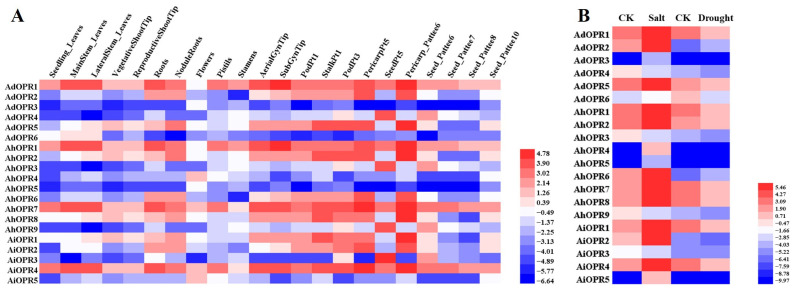
Expression profiles of *OPR* genes in 22 different tissues and stress conditions. (**A**) Heatmap of *OPR* family genes in 22 peanut tissues; (**B**) heatmap of *OPR* family genes under salt and drought treatments. Fragments per kilobase of transcript per million fragments (FPKM) of peanut *OPR* genes were log2-transformed using Heml_1.0 software.

To gain more insights into the temporal and spatial expression patterns of *OPR* genes, the RNA sequences of different treatments under abiotic stress (Table S5) and 22 tissues (Table S6) were analyzed. Most genes exhibited different expression levels across 22 tissues (Figure 6A). The results show that four genes had relatively high expression levels in all 22 tissues, while three genes had relatively low expression levels in all 22 tissues (Figure 6A). Some peanut *OPR* genes exhibited tissue-specific expression patterns, such as *AhOPR4* and *AiOPR5*, which were prominently expressed in flowers. Additionally, *AdOPR4*, *AhOPR3*, *AhOPR9*, and *AiOPR3* displayed specific expression in the seeds.

The expression levels of the genes under drought and salt stresses were investigated using our published transcriptome sequencing results. We found that the expression pattern of peanut *OPR*s was notably different under drought and salt stresses. In Figure 6B, the expression of most peanut *OPR*s (16 of 20) was upregulated by more than 2-fold under salt stress, and the majority of *OPR*s were increased by up to 10-fold. Unlike salt treatment, only three *OPR*s (*AdOPR2*, *AhOPR6*, and *AiOPR3*) were upregulated under drought stress, and the majority *OPR* genes were downregulated. It is worth noting that the expression of four *OPR* genes (*AdOPR3*, *AhOPR4*, *AhOPR5*, and *AiOPR5*) was unchanged in response to drought stress. Only two genes (*AdOPR2* and *AhOPR6*) were upregulated both under salt and drought stresses, and the *AhOPR6* gene was selected for further functional study.

### 2.7. Subcellular Location of Peanut OPR Proteins

**Figure 7:**
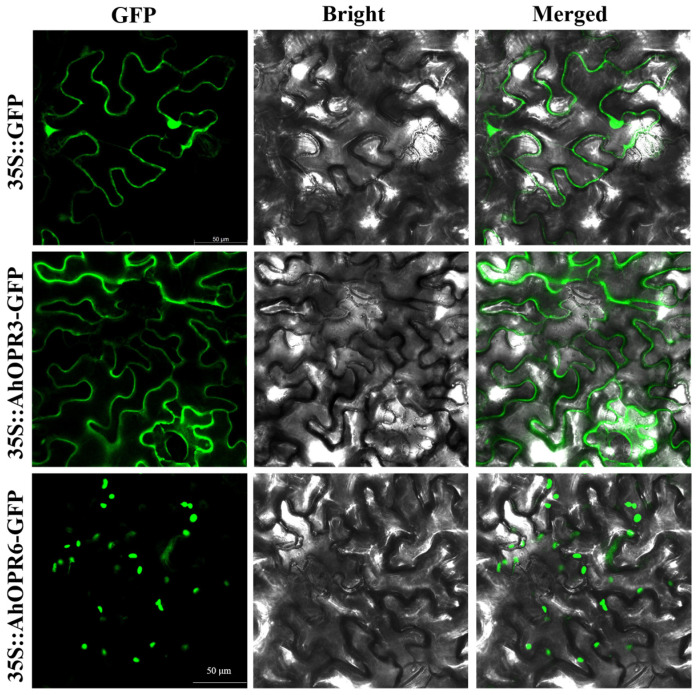
The subcellular localization of *AhOPR3* and *AhOPR6* proteins. The *AhOPR3*-GFP, *AhOPR6*-GFP fusion protein, and GFP protein were transiently expressed into tobacco epidermal cells. Bar = 50 μm.

*AhOPR* proteins were predicted to be localized in the chloroplast, plasma membrane, or mitochondria using the online web tool WoLF PSORT (Table S1). In order to confirm the subcellular localization of the peanut proteins, *AhOPR3* and *AhOPR6* proteins were selected to perform the experiment (Figure 7). The *35S::AhOPR3-GFP* and *35S::AhOPR6-GFP* fusion proteins were constructed, together with 35S::GFP (the empty vector), and they were transiently expressed in *N. benthamiana*. Confocal microscopy observations revealed that the green fluorescence of the empty vector was found in the cytoplasm and on the plasma membranes, while the *35S::AhOPR3-GFP* fusion protein was distributed on the plasma membranes, and the 35S::*AhOPR6*-GFP fusion proteins was localized in the chloroplast (Figure 7).

### 2.8. Gene Ontology (GO) and Interactive Networks of Peanut OPR Genes

**Figure 8:**
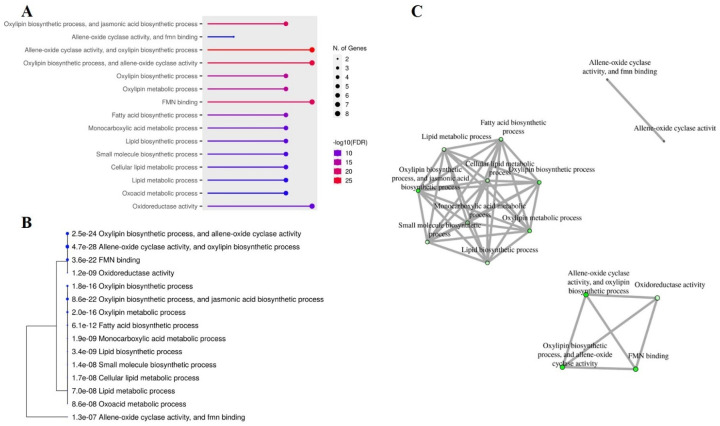
Analysis of gene ontology (GO) and interactive enrichment networks in ShinyGO database. (**A**) GO of *AhOPR6* genes; (**B**) phylogenetic analysis of *AhOPR6* gene annotations; (**C**) interactive enrichment networks of *AhOPR6* genes.

GO analysis offers valuable information for investigating potential associations based on their enrichment in molecular function (MF), cellular components (CCs), and biological process (BP). This analytical tool is commonly used in genetics to validate the functions of candidate genes. As shown in Figure 8A, all peanut *OPR*s were significantly enriched to allene oxide cyclase activity (MF), FMN binding (MF), the oxylipin biosynthetic process (BP), the jasmonic acid (JA) biosynthetic process (BP), and the oxylipin metabolic process (BP). All *OPR* genes were found to be associated with allene oxide cyclase activity, the oxylipin biosynthetic process, and FMN binding (Figure 8B). A further annotation analysis of *AhOPR*s using data from ShingGO confirmed a tree of three clusters according to a correlation analysis (Figure 8C).

### 2.9. Overexpression of AhOPR6 Enhanced Salt Tolerance in Seedlings

**Figure 9:**
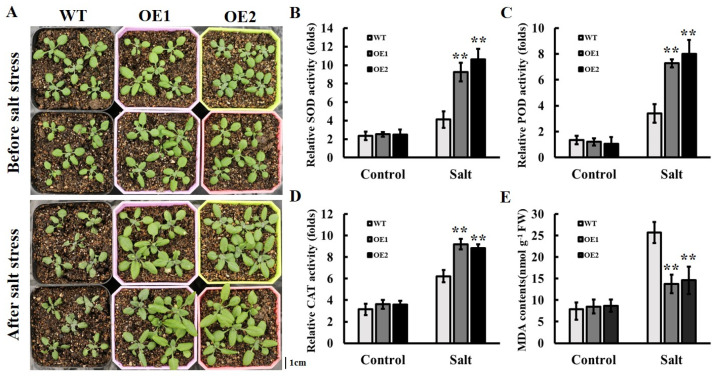
Overexpression of *AhOPR6* enhanced salt tolerance in *Arabidopsis*. (**A**) Phenotype of wild-type (WT) and OE lines with and without salt treatment. Bar = 1 cm. SOD (**B**), POD (**C**), and CAT (**D**) activities of overexpressing lines and WT Arabidopsis after salt stress. (**E**) MDA contents. Data are shown as means ± SD from three independent replications. ** *p* < 0.01 (*t*-tests).

To further explore the function of the *OPR* genes in salt response, overexpressing *AhOPR6* transgenic plants were investigated. Overall, twenty T0 transgenic lines were obtained by PCR using *Arabidopsis* genomic DNA. After cultivation and screening, the homozygous T3 transgenic lines of *AhOPR6* were used to analyze the functional roles in salt stress. After being treated with 300 mM of NaCl for one week, most leaves of the wild-type plants were wilted, while only a small number of OE1 and OE2 leaves became wilted (Figure 9A). Furthermore, the activities of SOD (Figure 9B), POD (Figure 9C), and CAT (Figure 9D) enzymes associated with ROS scavenging were increased after salt stress, and gene-overexpressed plants were obviously increased in SOD, POD, and CAT activity compared with wild-type *Arabidopsis*. Before the salt treatment, the wild-type and transgenic *Arabidopsis* lines had nearly equal MDA contents (Figure 9E). Transgenic *Arabidopsis* lines have significantly lower MDA contents after salt treatment (Figure 9E). These results reveal that the overexpression of the peanut *AhOPR6* gene enhanced salt tolerance in *Arabidopsis*.

### 2.10. The Overexpression of AhOPR6 in Arabidopsis Affects the Expression of JA/ABA Pathway-Related Genes

**Figure 10:**
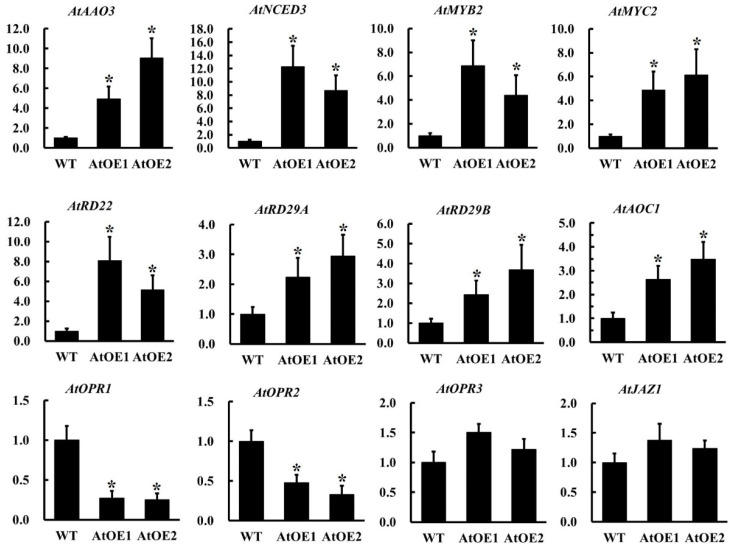
The relative expression level of JA/ABA pathway-related genes in the WT and OE lines of *Arabidopsis*. The data are shown as the means ± SD from three independent replications. * *p* < 0.05 (*t*-tests).

To further validate the role of ABA- and JA-related genes in *AhOPR6*-mediated salinity stress enhancement, the transcript abundance of 12 genes was monitored (Figure 10, Table S7). Some genes, like *AtAAO*, *AtNCED*, *AtRD*, *AtMYB*, and *AtMYC*, were key factors in the ABA-dependent stress-responsive signaling pathway; *AtAOC*, *AtOPR*, and *AtJAZ* were all key components of the JA biosynthesis pathway in *Arabidopsis*. The transcript abundance of *AtAAO3* and *AtNCED3* was increased in the AtOE-*AhOPR6* lines; *AtRD22*, *AtRD29A*, and *AtRD29B* were also upregulated in the *AhOPR6*-overexpressing AtOE lines (Figure 10). The expression levels of two upstream transcription factors, *AtMYB2* and *AtMYC2*, were significantly upregulated as expected. In the JA synthesis pathway, the expression levels of *AtAOC1*, *AtOPR1*, *AtOPR2*, and *AtJAZ1* were significantly upregulated in transgenetic *Arabidopsis* lines, while the expression of *AtOPR3* was not influenced by the overexpression of peanut *AhOPR6*.

## 3. Discussion

Peanut is an important economic and oil crop worldwide. However, annual worldwide losses caused by various biotic and abiotic stresses are destructive. The objective of this study was to explore all the OPRs in peanut and the potential role of OPRs in peanuts’ resistance. *OPR*s are key enzymes that participate in the last step of JA synthesis. JA and its derivates regulate the development and response to biotic and abiotic stresses in plants [7, 8, 30]. So far, the identification and functional analysis of the *OPR* gene family have been characterized in many plants, such as *Arabidopsis* (3), rice (12), tomato (4), maize (8), and wheat (48). However, the *OPR* gene family in cultivated peanut and its functional characterization have not been investigated. In this study, we constructed a rooted maximum likelihood (ML) phylogenetic tree with 20 peanut *OPR* genes in accordance with the phylogenetic relationships of two species, *Arabidopsis* and rice. Accordingly, the *OPR* gene family in peanut can be divided into two subfamilies (I and II). The number of *AhOPR* genes (9) is substantially higher compared to most diploid plants but lower than that of hexaploid wheat (48), which might be related to the fact that the allotetraploid genome of cultivated peanut has undergone whole-genome duplication and other gene duplication events [31,32]. There were six *AdOPR* genes in diploid peanut *A. duranensis* and five Ai*OPR* genes in diploid peanut *A. ipaensis*, while the number of allotetraploid *AhOPR* genes was less than the sum of two diploid *OPR* gene numbers. This phenomenon could be explained by reports that some homologous genes might have been lost or that some functionally redundant genes disappeared during the polyploidization of the genome [33,34].

Structure diversity within gene families was a mechanism for the evolution of many gene families. Intron gain and loss play a significant role in structural diversity and complexity. We found that most *OPR*s in peanut contain 3–5 extrons; all the genes in OPRII contain 4 introns, while most *OPR*s (approximately 70%) in OPR I have 2–3 introns, and the others contain more than 4 introns. In wheat, the *OPR* genes in subfamilies I, II, IV, and V contain 3–5 introns; 29 of 32 *OPR* genes in subfamily III contain 1–2 introns. In addition, subfamily III was the youngest subfamily in wheat which possessed the fewest introns [15]. The result was consistent with a previous study showing that the intron loss events occurred during the structural evolution of the *OPR* gene family [14]. Analogously, four of six maize *OPR* genes in subgroup I (*ZmOPR1*-*6*) contain 0–1 introns; the other two genes in subgroup II (*ZmOPR7* and *ZmOPR8*) have 3 or 4 introns [19]. The above results indicate that intron loss events could be found in different plants, whether dicots or monocots. All peanut *OPR*s in subfamilies I and II had motif 1, motif 2, motif 3, motif 5, and motif 7. These five motifs were related to the most conserved domains of *OPR*s and were associated with the alpha or beta barrel of protein secondary structures. Interestingly, the position of motif 9 of three genes (*AiOPR3*, *AiOPR5*, and *AdOPR3*) was replaced with motif 4. Four *OPR*s in OPRII lacked motif 8, which would impact the β6 barrel of the protein secondary structures. Structural integrity is essential to ensuring gene function, and slight alterations in the DNA structure or protein motifs usually affect gene functions [35,36].

Cis-acting elements are found in the promoter regions upstream of protein-coding genes, and they are transcription factor (TF) binding sites. TFs and cis-acting elements interact to control the precise initiation of gene transcription, resulting in tissue-specific and signal-specific gene expression [37]. Many cis-acting elements related to growth and development (such as endosperm and meristem development), hormones (including MeJA, ABA, SA, GA, and auxin), and stresses (such as drought, zein metabolism, low-temperature, circadian, anaerobic, and defense responses) have been identified in peanut *OPR* promoters. Among hormone-responsive elements, MeJA responsiveness cis-acting elements (TGACG-motif and CGTCA-motif) were the most abundant, followed by ABA-responsive element ABRE, SA-responsive TCA element, and the auxin-responsive TGA element. Moreover, MeJA responsiveness has been demonstrated to be linked to plant defense mechanisms [38]. Therefore, we can infer the function of peanut *OPR* genes through the cis-acting elements in the gene promoter, which offered an efficient method for screening potential candidate genes.

The stationary nature of plants makes them susceptible to a variety of stresses, and peanut is no exception. Various abiotic stresses and biotic stresses, like stress caused by salinity, drought, cold, heat, and herbivores, adversely impact staple food crops’ survival, biomass production, and yield by up to 70% [39,40,41]. According to recent research, drought affects approximately 45% of all agricultural land in the world, while salt impacts about 19.5% of it [42]. In accordance with the FAO’s estimates, salinity affects over 6% of the world’s land [41]. According to recent studies, *OPR*s were positively regulated in *Arabidopsis*, maize, and wheat when exposed to salt stress [22,43]. In maize, the overexpression of *ZmOPR1* affected *RD22*, *RD29B*, and *ABF2*, which are genes associated with osmotic and NaCl stresses [43]. *RD22* and *RD29B* are ABA-responsive genes; *ABF2* belongs to the ABF subfamily of bZIP proteins that interact with ABA-responsive elements. They are involved in ABA/stress responses [44,45,46]. Likewise, wheat *OPR1* could be induced by ABA and salinity, and the inhibition of ABA synthesis largely counteracts salinity-induced *TaOPR1* accumulation. As a result, there is a convincing argument that *TaOPR1* expression is induced by ABA synthesis. *AtRD22*, *AtRD29A*, and *AtRD29B* are all upregulated via an ABA-induced signaling cascade [47]. As a result of *TaOPR1* expression, *AtNCED3* and *AtAAO3*, which encode enzymes essential for ABA synthesis, were also significantly increased in transcript abundance. *AtMYB2*, an R2R3-MYB transcription factor gene, was induced by ABA, dehydration, and salt stresses [48,49], indicating that *AtMYB2* responds to environmental stresses through ABA-dependent and ABA-independent pathways [50,51]. Aside from *AtMYB2*, *AtMYC2* also activates genes in the ABA signaling pathway [52,53]. It has been documented that *AtMYB2* and *AtMYC2* can bind to MYB and MYC recognition sites in the promoter sequence of the *rd22BP1* gene, respectively, to regulate the expression of ABA-inducible genes (like *rd22*), thus enhancing transgenic plant stress resistance [53,54]. The expression of *AhOPR6* markedly increased the transcript abundance of *AtAAO3*, *AtNCED3*, *AtRD22*, *AtRD29A*, *AtRD29B*, *AtMYB2*, and *AtMYC2* (Figure 10). The above investigations provide firm evidence that *AhOPR6* plays a positive role in salt stress through ABA-dependent signaling pathways.

There are several factors that lead to salt stress on plants, including ion toxicity [55], osmotic stress [56], and reactive oxygen species (ROS) [57]. Salt stress leads to the accumulation of ROS such as superoxide, H_2_O_2_, and hydroxyl radicals; oxidative damage and ROS accumulation are both increased with prolonged salt stress exposure [58]. Similarly, other studies supported our results, indicating that significantly higher antioxidant enzyme activity reduced ROS production and led to improved osmotic adaptation to salt stress [59]. POD plays an important role in ROS signaling and redox reactions, and a high level of POD can increase plants’ salt tolerance [60]. In addition, the activities of POD and CAT were increased to reduce ROS accumulation under salt stress [61], and this result is consistent with our research. MDA, a product of membrane peroxidation, is often used as a prime indicator of oxidative stress in salt-stressed tissues [62,63]. Salt-sensitive plants exhibit a greater amount of MDA than salt-tolerant ones [64]. In our study, the decrease in MDA contents in AtOE lines implied that the overexpression of *AhOPR6* increased the plants’ salt tolerance mainly by reducing ROS damage.

**Figure 11:**
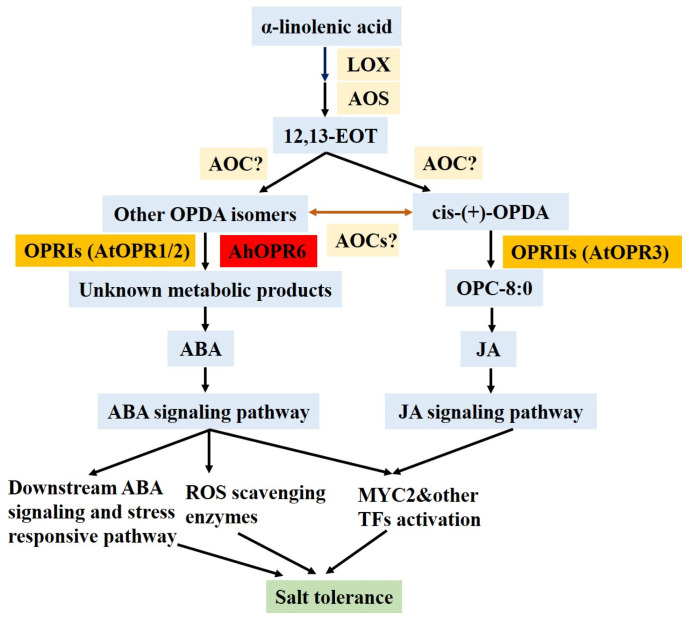
Salt stress signal and regulatory mechanism of *AhOPR6.*

JA and its intermediates are critical elements of perception and signaling, and JA-mediated gene expression and signaling pathways can be triggered by several corroborative receptors, activators, transcription factors, repressors, and co-repressors [8,10]. In the first half of JA biosynthesis, *LOX*, *AOS*, and *AOC* sequentially catalyze to generate the intermediate product cis-(+)-OPDA [65]. The expression levels of *AtOPR3* and *AtJAZ1* (receptor genes of JA) were unchanged (Figure 10); these results indicate that JA synthesis in OE lines does not depend on *AhOPR6*. Through *AtMYC2*, the JA-activated MKK3-MPK6 signal negatively regulates the JA pathway and affects JA-dependent gene expression [66]. It is worth noting that the bHLH transcription factor *AtMYC2*, activated by JA, significantly interacts with the signaling pathways of ABA, JA, GA, and ethylene [67]. We found that the expression level of *AtMYC2* was increased, but *AtJAZ1* was unchanged in AtOE-*AhOPR6* lines. These results could be attributed to the promotion of ABA but not the JA signaling pathway, and it is highly possible that *AhOPR6* was not regulating JA synthesis (Figure 11). The research conducted by Dong et al. also verified our hypothesis [22] that the OPRI (*TaOPR1*) gene exerts no regulatory activity on JA synthesis and JA signaling pathways.

## 4. Materials and Methods

### 4.1. Identification of Peanut OPR Gene Family Members

The protein sequence, gene annotation information file (gff3), and coding sequence (CDS) of the newly published peanut genome were obtained from the PeanutBase database (https://www.peanutbase.org/, accessed on 21 January 2022). The Hidden Markov model of the *OPR* gene family (PF00724) was downloaded from the Pfam database (https://pfam.xfam.org/, accessed on 5 May 2025) [68], and then this profile was used to search the peanut protein database with E-values < 1.2 × 10^−28^ by HMMER 3.0. The protein sequence of *OPR*s was extracted via Perl language and submitted to the web tools SMART (http://smart.embl.de/smart/batch.pl, accessed on 5 May 2025), Pfam (https://pfam.xfam.org/search, accessed on 5 May 2025), and NCBI-CDD (https://www.ncbi.nlm.nih.gov/cdd/, accessed on 5 May 2025) to verify the structural integrity of *OPR*. Finally, reliable *OPR* candidate genes were determined. The molecular weight (MW), amino acid sequence length (aa), and isoelectric point (pI) of the *OPR* were predicted using the online server ExPASY (https://web.expasy.org/cgi-bin/protparam/protparam, accessed on 5 May 2025) [69]. Subcellular localization of *OPR*s was predicted using the online web tool WoLF PSORT (https://wolfpsort.hgc.jp/, accessed on 5 May 2025).

### 4.2. Phylogenetic Analysis

To further understand the evolutionary relationship of the *OPR* gene family, 3 *Arabidopsis OPR*s and 11 rice *OPR*s amino acid sequences were downloaded from the NCBI database, and 20 peanut *OPR*s were downloaded from PeanutBase (https://www.peanutbase.org/, accessed on 5 May 2025). The multiple sequence alignment of amino acid sequences was conducted via the ClustalW function of MEGA X software, and the phylogenetic relationships of the three species were constructed using the neighbor-joining method; the bootstrap value was set to 1000. The classifications of *OPR*s in peanut were based on the topological structure of the phylogenetic tree and sequence similarity. The established phylogenetic trees were beautified using Evolview (http://www.evolgenius.info/evolview/, accessed on 5 May 2025) [70].

### 4.3. Analysis of Gene Structure and Protein Conserved Domains of OPRs

Based on peanut genome annotation information, location information of the exon, intron, and UTR of *OPR* genes on chromosomes was obtained, which was used to draw gene structure. Conserved motifs with lengths of 6–100 aa in 20 *OPR* proteins were analyzed using the MEME https://meme-suite.org/meme/tools/meme, accessed on 5 May 2025) program [71]. The maximum number of motifs was set at 10. The gene structure and conserved motifs of *OPR*s were visualized using TBtoolsV1.098 software [72].

### 4.4. Cis-Acting Regulatory Elements and Gene Ontology (GO) Annotation

The 2 kb promoter sequence of *OPR*s was extracted from peanut genomic DNA sequences of the PeanutBase database and uploaded to PlantCARE (http://bioinformatics.psb.ugent.be/webtools/plantcare/html/, accessed on 5 May 2025) [73] to predict cis-acting regulatory elements in the promoter region. The GO analysis was conducted in the ShinyGO 0.80 database (http://bioinformatics.sdstate.edu/go/, accessed on 5 May 2025) by inputting all *OPR* family genes to ascertain the molecular functions, biological processes, and cell components related to peanut *OPR*s [74].

### 4.5. Chromosome Localization and Synteny Analysis

To determine the distribution of *OPR*s on chromosomes, we obtained the starting and ending positions of *OPR* genes from peanut genome annotation files (gff3) and submitted their location information to the online tool MapGene2Chrom web v2 (http://mg2c.iask.in/mg2c_v2.0/, accessed on 5 May 2025) to map their chromosome positions. The segmental and tandem repeats of *OPR* family members in peanut were analyzed via MCScanX (http://chibba.pgml.uga.edu/mcscan2/, accessed on 5 May 2025) software [75]. Two adjacent genes located on the same chromosome with a relative position less than 100 kb are defined as tandem repeat genes [76]. The chromosome distribution of 20 *OPR* genes and the collinearity of related genes were plotted by using Circos (version 0.69) software [77].

### 4.6. RNA-Seq Data Analysis of OPRs

The expression patterns of *OPR* genes in different tissues and different stress treatments were investigated in peanut. In this study, RNA-seq data of 22 tissues were derived from the PeanutBase database [78], and the FPKM values of drought [79] and salt [80] stresses were obtained from our previous work. The PFKM values of *OPR* family genes were standardized by log2 PFKM, and the heatmap was drawn using Heml_1.0 software.

### 4.7. Subcellular Localization of AhOPR6 Protein

The coding sequences of *AhOPR3* and *AhOPR6* were amplified from peanut cultivar “huayu71” and used to construct the *35S::AhOPR3-GFP* and *35S::AhOPR6-GFP* fusion vectors. The empty vector and the two fusion vectors were transiently expressed in *Nicotiana benthamiana* leaves. After three days, a confocal microscope (TCS-SP8, Leica, Wetzlar, Germany) was used to observe the fluorescence signals of the fusion proteins in the cells.

### 4.8. Construction of AhOPR6 Vectors and Generation of Transgenic Arabidopsis Plants

Full-length coding sequence of *AhOPR6* was amplified from the cDNA of peanut cultivar “huayu71” by PCR. To construct the overexpression vector, the *AhOPR6* gene was placed between the 35S promoter and the Nos terminator in the pCambia2300EC vector. The recombinant plasmid was transformed into *Agrobacterium* strain GV3101, which was then applied in floral dipping to transform wild-type *Arabidopsis*. Positive seedlings were derived from the selective media after being grown on MS media with 50 mg/l kanamycin, and T3 homozygous seedlings were employed for subsequent analysis.

### 4.9. The Treatment and Determination of Salt-Related Physiological Parameters in Transgenic Arabidopsis

Both WT and transgenic *Arabidopsis* seedlings were grown on 1/2 MS media for 7 days and then transferred to soil for 2 weeks. For salt stress, *Arabidopsis* seedlings were treated with 300 mM NaCl for one week. The malondialdehyde (MDA) content, superoxide dismutase (SOD), peroxidase (POD), and catalase (CAT) enzyme activities were detected using assay kits (Comin, Suzhou, China) for both overexpressing lines and WT *Arabidopsis*. The four physiological parameters of the *Arabidopsis* plants were measured in triplicate.

### 4.10. RNA Isolation and Real-Time Quantitative PCR (qRT-PCR) Validation of OPR

Trizol reagent (TaKaRa, Dalian, China) was used to extract total RNA from leaves of *Arabidopsis* under salt stress treatments. The integrity and quality of RNA were detected using 1% agarose gel electrophoresis, and the concentrations of RNA, namely OD260/280 and OD260/230, were detected using the Nanodrop one micro-ultraviolet spectrophotometer. Total RNA was reverse-transcribed into cDNA using the Transcriptor First Strand cDNA Synthesis Kit (TaKaRa, Dalian) and stored at −20 °C. qRT-PCR was performed on ABI7500 (Applied Biosystems, Foster City, CA, USA). The reaction system was as follows: 10 μL of 2×UltraSYBR Mixture, 0.4 μL of forward primer (5.0 μmol/L), 0.4 μL of reverse primer (5.0 μmol/L), 1 μL of cDNA (200 ng), and 7.4 μL of RNase-free water. The amplification procedure was as follows: 95 °C for 5 min, 40 cycles of 95 °C for 10 s, and 60 °C for 1 min; 95 °C for 15 s, 60 °C for 1 min, 95 °C for 15 s, and 60 °C for 15 s. Three biological repeats and three technical repeats were set up in each treatment. The relative expression of genes was calculated using 2^−ΔΔCt^ [81]. The fluorescent quantitative primers were designed using Primer-BLAST (https://www.ncbi.nlm.nih.gov/tools/primer-blast/index.cgi, accessed on 5 May 2025).

## 5. Conclusions

In this study, a total of 20 *OPR* genes were identified in the genome of a tetraploid cultivar and two diploid peanut species. They were divided into two subfamilies with *Arabidopsis* and rice homologous genes. The investigation of the evolutionary relationship, gene structure, protein conserved motifs, chromosome locations, gene duplication, tissue expression patterns, and expression patterns under salt and drought stresses indicated their diversity in different *OPR* genes. The subcellular location of peanut *AhOPR3* and *AhOPR6* revealed that they were distributed on the plasma membranes and localized in the chloroplast, respectively. Furthermore, the overexpression of *AhOPR6* in Arabidopsis increased tolerance to salt stress. Notably, the detection of JA and ABA signaling pathway-related genes by qRT-PCR indicated that *AhOPR6* might enhance salt stress tolerance through an ABA-dependent pathway. Overall, we constructed a hypothetical model of *AhOPR6*’s action in improving salt tolerance and provided candidate *OPR* genes for further functional studies in growth and development, as well as peanut stress resistance breeding.

## Data Availability

The original contributions presented in this study are included in the article/supplementary material. Further inquiries can be directed to the corresponding author(s).

## Supplementary Materials

The following supporting information can be downloaded at https://www.mdpi.com/article/10.3390/plants14101408/s1. Table S1: Characteristics of putative peanut *OPR* genes; Table S2: Detailed information about conserved motifs of *AhOPR*s; Table S3: Tandem and segmentally duplicated *OPR* gene pairs in peanut; Table S4: Analysis of cis-acting regulatory elements of peanut *OPR* promotors; Table S5: FPKM values of peanut *OPR* genes under two abiotic stresses (drought and salt) in this study; Table S6: FPKM values of peanut *OPR* genes in 22 tissues in this study; Table S7: Primer sequences used in this study; Figure S1: Three-dimensional structure prediction of peanut *OPR*s.
