# Supplementary figures and images for: Genome-Wide Analysis of the 12-Oxo-Phytodienoic Acid Reductase Gene Family in Peanut and Functional Characterization of AhOPR6 in Salt Stress
Source: Plants (Basel). 2025 May 8;14(10):1408. doi: 10.3390/plants14101408 (PMC12114951; doi:10.3390/plants14101408)

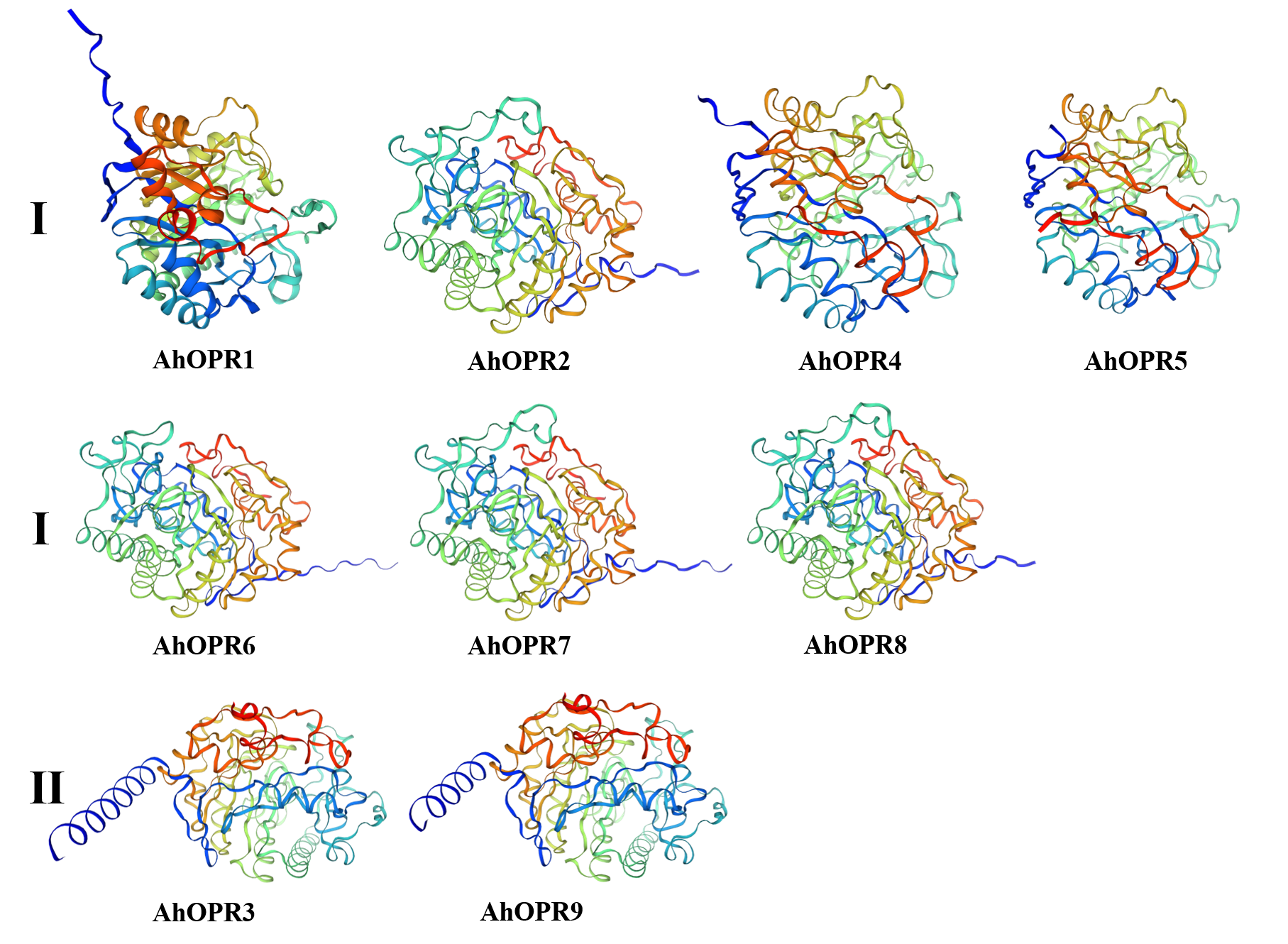

Supplement: Supplementary file 1 [file plants-14-01408-s001.zip › Fig S1.png]
